# Smc5/6 in the rDNA modulates lifespan independently of Fob1​

**DOI:** 10.1111/acel.13373

**Published:** 2021-05-12

**Authors:** Sarah Moradi‐Fard, Aditya Mojumdar, Megan Chan, Troy A.A. Harkness, Jennifer A. Cobb

**Affiliations:** ^1^ Departments of Biochemistry & Molecular Biology and Oncology Robson DNA Science Centre Arnie Charbonneau Cancer Institute Cumming School of Medicine University of Calgary Calgary AB Canada; ^2^ Department of Biochemistry, Microbiology and Immunology University of Saskatchewan Saskatoon SK Canada

**Keywords:** Fob1, nucleolar morphology, nucleolus, rDNA, replicative lifespan, silencing, Smc5/6

## Abstract

The ribosomal DNA (rDNA) in *Saccharomyces* *cerevisiae* is in one tandem repeat array on Chromosome XII. Two regions within each repetitive element, called intergenic spacer 1 (IGS1) and IGS2, are important for organizing the rDNA within the nucleolus. The Smc5/6 complex localizes to IGS1 and IGS2. We show that Smc5/6 has a function in the rDNA beyond its role in homologous recombination (HR) at the replication fork barrier (RFB) located in IGS1. Fob1 is required for optimal binding of Smc5/6 at IGS1 whereas the canonical silencing factor Sir2 is required for its optimal binding at IGS2, independently of Fob1. Through interdependent interactions, Smc5/6 stabilizes Sir2 and Cohibin at both IGS and its recovery at IGS2 is important for nucleolar compaction and transcriptional silencing, which in turn supports rDNA stability and lifespan.

## Graphical abstract

Smc5/6 is important for transcriptional silencing in the rDNA. rDNA compaction requires Smc5/6 localization to IGS regions. Transcriptional silencing of ncRNA at IGS1 and IGS2 is differentially regulated. Smc5/6 has a role in rDNA maintenance independent of HR processing at the RFB. Fob1‐independent disruption of Smc5/6 leads to lifespan reduction.

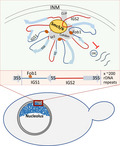

## 1 INTRODUCTION

**FIGURE 1:**
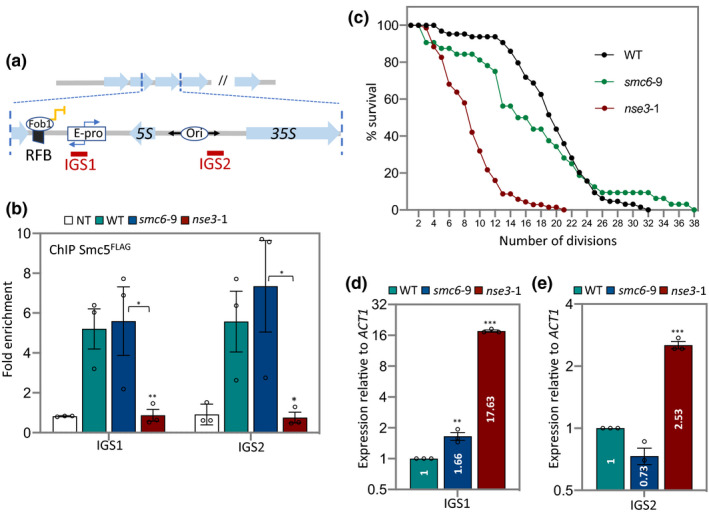
Smc5/6 localization to IGS1 and IGS2 is important for lifespan. (a) Schematic of rDNA repeats in *Saccharomyces cerevisiae* showing non‐transcribed spacers (IGS1 and IGS2) flanking the transcribed 5S and 35S sequences in one repeat. The location of primer sites used in ChIP experiments are illustrated. (b) Enrichment of Smc5^FLAG^ at IGS1 and IGS2 by ChIP with α ‐FLAG antibody in non‐tagged control (JC 470), WT (JC 3728), *smc6‐9* (JC 5894) and *nse3‐1* (JC 5879) at IGS1 and IGS2. Fold enrichment is based on normalization to negative control region as described in the experimental procedures. (c) Replicative lifespan measured and represented as percentage of survival of mother cells with each division for WT (JC 471), *smc6‐9* (JC 1358) and *nse3*1 (JC 3032) strains. (d and e) Transcription at (d) IGS1 and (e) IGS2 relative to WT cells after normalization to *ACT1* transcription for WT (JC 471), *smc6‐9* (JC 1358) and *nse3‐1* (JC 3032). Analysis was performed using at least three biological replicates. Asterisks indicate statistical significance versus WT unless otherwise noted. Statistical analysis is described in Section 4

The ribosomal DNA in *Saccharomyces* *cerevisiae* (budding yeast) consists of approximately 150–200 identical 9.1 kb long tandem repeats on chromosome XII which are assembled in one cluster and positioned close to the nuclear periphery (Gartenberg & Smith, 2016). The 35S and 5S ribosomal RNA genes are transcribed by RNA polymerases I and III, respectively. Intergenic spacer 1 and 2 (IGS1 and IGS2) regions flank the rRNA genes and are usually silenced, however, they can be transcribed by RNA polymerase II to produce noncoding (nc) RNAs (Figure 1a; Bryk et al., 1997; Smith & Boeke, 1997). IGS1 contains a replication fork barrier (RFB) sequence and a bi‐directional non‐coding promoter, called E‐pro and IGS2 contains the autonomous replication sequence (ARS) element used as the start site for replication in the rDNA. The histone deacetylase Sir2 interacts with Net1 and Cdc14 in the nucleolus to form the RENT complex, which represses transcription from IGS1 and IGS2 (Bryk et al., 1997; Fritze et al., 1997; Gottlieb & Esposito, 1989; Huang et al., 2006; Imai et al., 2000; Li et al., 2006; Shou et al., 1999; Smith & Boeke, 1997; Straight et al., 1999; Vasiljeva et al., 2008; Visintin et al., 1999). The recovery of Sir2 at IGS2 appears to be dynamic and dependent on RNA Polymerase I transcription of 35S, which is found at ~50% of rDNA genes in asynchronously growing cell cultures (Huang & Moazed, 2003; Li et al., 2013). The recovery of Sir2 at IGS1 is through another mechanism that has been characterized more extensively compared to its binding at IGS2. At IGS1, the Fob1 protein binds the RFB, ensuring unidirectional replication and the localization of RENT, which represses E‐pro transcription. When the progressing replication fork is stalled by Fob1, a double strand break (DSB) results and it is repaired by recombination between the repetitive sequences (Brewer & Fangman, 1988; Kobayashi, 2003; Kobayashi et al., 1992). From an evolutionary perspective, the events at IGS1 are important to maintain rDNA copy number. The DSB can be repaired through unequal sister chromatid recombination (USCR), allowing changes in the number of repetitive elements (contraction or expansion; Johzuka & Horiuchi, 2002). Increased transcription from the E‐pro, loosens chromatin adjacent to the RFB‐induced DSB, which in turn leads to increased USCR‐mediated repair. If this highly dynamic process is not tightly regulated, then rDNA instability arises. For example, the deletion of *SIR2* results in transcription from IGS1. However, most of the down‐stream consequences of *sir2*Δ depend on the formation of a DSB at the RFB and are reversed through the additional deletion of *FOB1*. (Kobayashi & Ganley, 2005; Saka et al., 2013).

The rDNA associates with the perinuclear membrane through Cohibin and the chromosome linkage INM proteins (CLIP) complexes. Cohibin, consisting of Csm1 and Lrs4, physically associates with the CLIP complex in order to keep the repetitive elements sequestered away from the HR machinery (Huang et al., 2006; Mekhail et al., 2008). Cohibin silences IGS1 independently of Sir2 and silencing and tethering defects are seen in *csm1*Δ or *lrs4*Δ mutants (Corbett et al., 2010; Huang et al., 2006; Mekhail et al., 2008; Rabitsch et al., 2003; Smith et al., 1999). By contrast, disruption of the CLIP complex by *HEH1* deletion does not affect silencing of rDNA although tethering is lost (Mekhail et al., 2008).

Recombination in the rDNA is influenced by multiple inter‐related mechanisms including chromatin condensation, transcriptional silencing and spatial organization, which is partly mediated by anchoring the repetitive elements at the inner nuclear membrane (Mekhail & Moazed, 2010). Moreover, related work shows that decreased rDNA stability correlates with reduced lifespan (Ganley et al., 2009; Henderson & Gottschling, 2008; Sinclair & Guarente, 1997). In yeast this is the number of times a mother cell can bud and give rise to daughter cells before it dies (Kennedy et al., 1995; Mortimer & Johnston, 1959; Muller et al., 1980). Cells where *SIR2* was deleted showed a decrease in lifespan and an increased production of extrachromosomal rDNA circles (ERCs). Early work suggested that ERCs were causative of premature senescence by titrating limited replication and transcription factors from the genome (Sinclair & Guarente, 1997). However, subsequent work suggested that rDNA instability itself drives aging with ERC accumulation being a correlation (Ganley & Kobayashi, 2014). Regardless, loss of fork pausing at the RFB in *fob1*Δ mutants has two effects, it prevents ERC formation and increases lifespan. The reduced lifespan of *sir2*Δ mutants was suppressed to wild type by deleting *FOB1*. However, the lifespan in *fob1*Δ single mutant cells is extended beyond wild type suggesting that Sir2 contributes to rDNA stability through a mechanism independent of Fob1 (Kaeberlein et al., 1999).

The Smc5/6 complex belongs to the structural maintenance of chromosome (SMC) family, which also includes cohesin and condensin (Jeppsson et al., 2014). Cohesin regulates cohesion between sister chromatids and condensin drives chromosome compaction by linking together different regions of the same chromosome. While the three SMC complexes are important for chromosome structure and organization, the involvement of Smc5/6 in higher level chromosome structure remains vague relative to cohesin and condensin. By contrast Smc5/6 has been studied more extensively in homologous recombination and DNA replication and it associates with repetitive regions of the genome, like the rDNA and telomeres, to resolve HR‐dependent intermediates (Lindroos et al., 2006; Menolfi et al., 2015; Torres‐Rosell et al., 2005). All components of the complex are essential for life including Smc5 and Smc6, and the six non‐Smc elements, Nse1‐6, with Nse2 most commonly referred to as Mms21. Investigating their functions in vivo has relied heavily on characterizing thermosensitive (ts) mutants, which limits our understanding of the complex to only a subset of functions (Menolfi et al., 2015; Peng et al., 2018; Torres‐Rosell, De Piccoli, et al., 2007; Torres‐Rosell et al., 2005). Cells harboring the *smc6‐9* allele display delayed rDNA replication, increased chromosomal breakage and accumulated X‐shaped DNA structures (Torres‐Rosell, De Piccoli, et al., 2007; Torres‐Rosell et al., 2005). Replication and HR‐related defects have also been reported using degron‐inducible mutants (Peng et al., 2018). The accumulation of HR intermediates in ts and degron‐tagged Smc5/6 complex mutants were reversed by deleting *FOB1* (Peng et al., 2018; Torres‐Rosell, De Piccoli, et al., 2007). These observations, together with other HR‐related investigations showed Smc5/6 to be integral for controlling Fob1‐dependent HR‐mediated processes at the rDNA (Kegel & Sjogren, 2010; Murray & Carr, 2008; Palecek, 2018).

Smc5/6 has been implicated in transcriptional silencing at the rDNA and telomeres in *S*. *pombe* and *S*. *cerevisiae*, respectively (Irmisch et al., 2009; Moradi‐Fard et al., 2016; Poon & Mekhail, 2011; van Ruiten & Rowland, 2018). However, a quantitative measurement of transcription throughout the rDNA array has not yet been demonstrated in budding yeast nor has the importance of Smc5/6 in lifespan been reported. Here we define a broader function for Smc5/6 in rDNA homeostasis by characterizing two mutant alleles, *smc6‐9* and *nse3‐1*, alone and in combination with canonical silencing factors (Moradi‐Fard et al., 2016). In *smc6‐9* mutants, the complex is HR deficient but localizes to the rDNA and in *nse3‐1* mutants the complex is not recovered there. This is similar to our earlier findings where we showed Smc5/6 assembles as a complex in both alleles but only localizes to telomeres in *smc6‐9*, but not *nse3‐1* mutants (Moradi‐Fard et al., 2016). Here we find that Smc5/6 is important for the binding of Cohibin and Sir2 at IGS1, while Fob1 and Sir2 are required for optimal binding of Smc5/6 to IGS1 and IGS2, respectively. In all, we demonstrate that Smc5/6 binding in the rDNA is important not only for HR processing at the RFB, but for IGS2 silencing, nucleolar compaction, and replicative lifespan.

## 2 RESULTS

### 2.1 Absence of the Smc5/6 complex at rDNA results in silencing defects and short lifespan

While rDNA stability correlates with lifespan and transcriptional silencing, the importance of Smc5/6 in lifespan has not been reported. Previous work with a ts allele of *SMC6*, *smc6‐9*, showed that the complex is important for processing HR intermediates that arise when replication forks stall at RFBs in IGS1 (Torres‐Rosell, De Piccoli, et al., 2007; Torres‐Rosell et al., 2005). Here we characterize *smc6‐9* and another ts allele, *nse3‐1*, which was previously shown to disrupt the localization of Smc5/6 to telomeres and to disrupt telomere clustering at the nuclear periphery (Moradi‐Fard et al., 2016). To determine Smc5/6 binding in the rDNA, we performed chromatin immunoprecipitation (ChIP) with Smc5^FLAG^ followed by qPCR with primers designed to IGS1 and IGS2 (Figure 1a). Similar to previous reports, Smc5^FLAG^ was enriched in the rDNA at both IGS sites (Figure 1b; Torres‐Rosell et al., 2005). The level of Smc5^FLAG^ in *smc6‐9* was similar to wild type, indicating that the rDNA defects previously reported with this allele do not stem from defects in complex binding. In contrast, there was a significant reduction of Smc5^FLAG^ recovered at IGS1 and IGS2 in *nse3‐1* mutant cells, to levels indistinguishable from the non‐tagged control (Figure 1b). Similar enrichment levels were observed in both alleles at 37°C too (Figure S1a). Not only was Smc5 reduced at IGS regions, but also at sites in the 35S and 5S ribosomal RNA genes in *nse3‐1* mutant cells, indicating that *nse3‐1* might impose a global DNA‐binding defect for the Smc5/6 complex in the rDNA (Figures S1b–d). This is consistent with what we previously observed where *nse3‐1* mutants showed reduced Smc5/6 recovery at telomeres leading to TPE and telomere clustering defects (Moradi‐Fard et al., 2016).

Factors involved in rDNA replication, transcription, and chromatin accessibility impact replicative lifespan. While Smc5/6 binds in the rDNA and has links with these processes, a potential role in lifespan has never been reported. Compared to wild type, cells harboring either mutant allele showed a reduced lifespan, however, it decreased more in *nse3‐1* than *smc6‐9* mutants (Figure 1c). We reasoned that a comparative analysis of *nse3‐1* and *smc6‐9* alleles could help identify functions for Smc5/6 that maintain lifespan, which could extend beyond its role in HR processing. As such, the levels of ncRNAs at IGS1 and IGS2 were measured in the mutant alleles. While there was a mild increase in transcription at IGS1, transcript levels remained low at IGS2 in HR deficient *smc6‐9* mutants (Figure 1e). Transcription was markedly higher at both sites in *nse3‐1* mutants, identifying a role for Smc5/6 in IGS2 silencing (Figure 1d,e).

### 2.2 Smc5/6 complex interacts with CLIP and is required for Heh1‐mediated rDNA tethering and Heh1‐independent rDNA compactness

**FIGURE 2:**
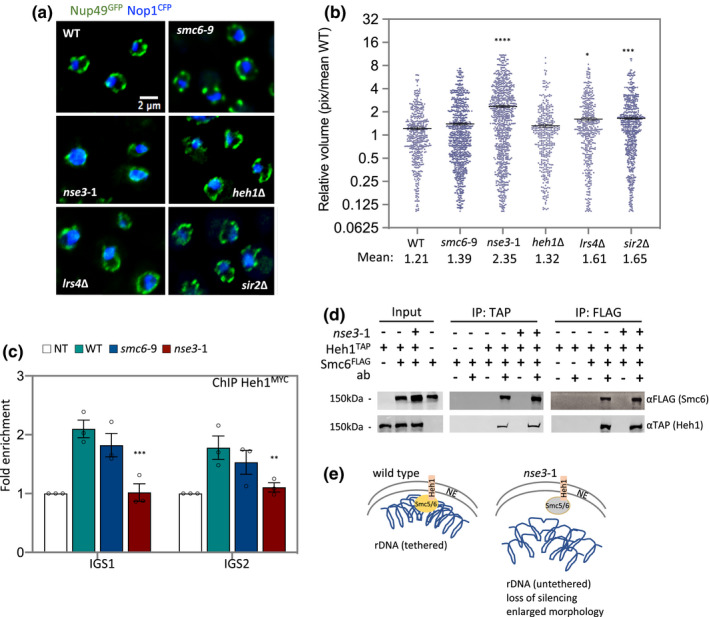
Smc5/6 tethers rDNA repeats at the periphery and interacts with Heh1. (a) Nucleolus morphology is illustrated by imaging CFP‐tagged NOP1 in WT (JC 4676), *smc6‐9* (JC 4932), *nse3‐1* (JC 4729), *heh1*Δ (JC 4735), *lrs4*Δ (JC 4731) and *sir2*Δ (JC 4633); GFP‐tagged NUP49 indicates nuclear periphery boundaries. (b) Scatter plot data of nucleolar volume for WT (JC 5016), *smc6‐9* (JC 5014), *nse3‐1* (JC 5015), *heh1*Δ (JC 4735), *lrs4*Δ (JC 4731) and *sir2*Δ (JC 4633) were measured in pixel and represented relative to mean of WT as described in Section 4. (c) Enrichment of Heh1^MYC^ at IGS1 and IGS2 by ChIP with α‐MYC in non‐tagged control (JC 470), WT (JC 4022), *nse3‐1* (JC 4228) and *smc6‐9* (JC 4942) at IGS1 and IGS2. Fold enrichment is represented as relative to no tag control after normalization to the negative control region described in Figure1. (d) Co‐IP between Smc6^FLAG^ and Heh1^TAP^ followed by western blotting with antibodies to epitope tags on each protein in the in negative controls (JC 1594; for α‐TAP IP) or (JC 4107, for α‐FLAG IP), WT (JC 4811) and *nse3‐1* (JC 4813) cells. (e) Schematic representation of Smc5/6 in rDNA tethering at the periphery in wild type and *nse3‐1* cells. Asterisks indicate statistical significance versus WT unless otherwise noted. Analysis was performed using at least three biological replicates. Statistical analysis is described in Section 4

Previous work in budding yeast showed that a reduced lifespan often correlates with increased nucleolar volume (Unal et al., 2011). Therefore, we visualized the morphology of the nucleolus in wild type and mutant cells. Nop1^CFP^ marked the nucleolus and Nup49^GFP^ marked the nuclear periphery (Figure 2a). In *smc6‐9* mutant cells, nucleolar volume remained largely unchanged (Figure 2b), whereas in *nse3‐1* mutant cells, the mean volume of the nucleolus was almost twice as large as wild type (Figure 2b), correlating Smc5/6 binding in the rDNA with nucleolar compaction (Figures 1b and 2b).

Given the enlarged nucleolar morphology in *nse3‐1* mutants and previous work showing Smc5/6 localizes to the nuclear periphery (Zhao & Blobel, 2005), we investigated a potential role for the complex in anchoring the rDNA to the inner nuclear membrane (INM). Heh1 and Nur1 reside in the INM and forms the CLIP complex. The recovery of Heh1 in the rDNA by ChIP has been used to measure anchoring of the repeats at the nuclear periphery (Mekhail et al., 2008). Compared to wild type, Heh1^MYC^ enrichment at IGS1 and IGS2 decreased significantly in *nse3‐1*, but not *smc6‐9* mutants (Figure 2c).

In contrast, the reverse experiment showed that the deletion of *HEH1* did not alter Smc5/6 enrichment in the rDNA (Figure S2). Interestingly, nucleolar volume in the absence of *HEH1* was indistinguishable from wild type cells (Figure 2a,b), indicating loss of tethering alone does not directly lead to increased nucleolar morphology.

Additional causes must contribute to the enlarged nucleolar volume in *nse3‐1* mutants, We next investigate whether Smc5/6 physically interacted with the CLIP complex. We performed co‐immunoprecipitations (IPs) between Heh1^TAP^ and Smc6^FLAG^ in wild type and *nse3‐1* mutant cells. Smc6^FLAG^ was recovered in α‐TAP (Heh1) pulldowns and vice versa, Heh1^TAP^ was recovered in α‐FLAG (Smc6) IPs (Figure 2d). Recovery was not noticeably altered in cells harboring the *nse3‐1* allele (Figure 2d). Taken together these data show that when Smc5 and Heh1 are not recovered at IGS1 and IGS2 in the rDNA by ChIP in *nse3‐1* mutants (Figures 1b and 2c), that Smc5/6 and CLIP still physically associate at a level indistinguishable from wild type (Figure 2e).

### 2.3 Cohibin and Sir2 recovery at IGS1 and IGS2 depend on Smc5/6 localization

Lrs4 and Csm1 form the Cohibin complex, which interacts with Sir2 as part of the RENT complex and both are silencing complexes interacting with CLIP to tether the repeats at the perinuclear membrane (Chan et al., 2011; Corbett et al., 2010; Huang et al., 2006; Mekhail et al., 2008; Rabitsch et al., 2003). In a side‐by‐side comparison with Smc5/6 mutants, the deletion of either *LRS4* or *SIR2* led to increased nucleolar morphology, however, the increase was below that measured in *nse3‐1* mutant cells (Figure 2a,b).

**FIGURE 3:**
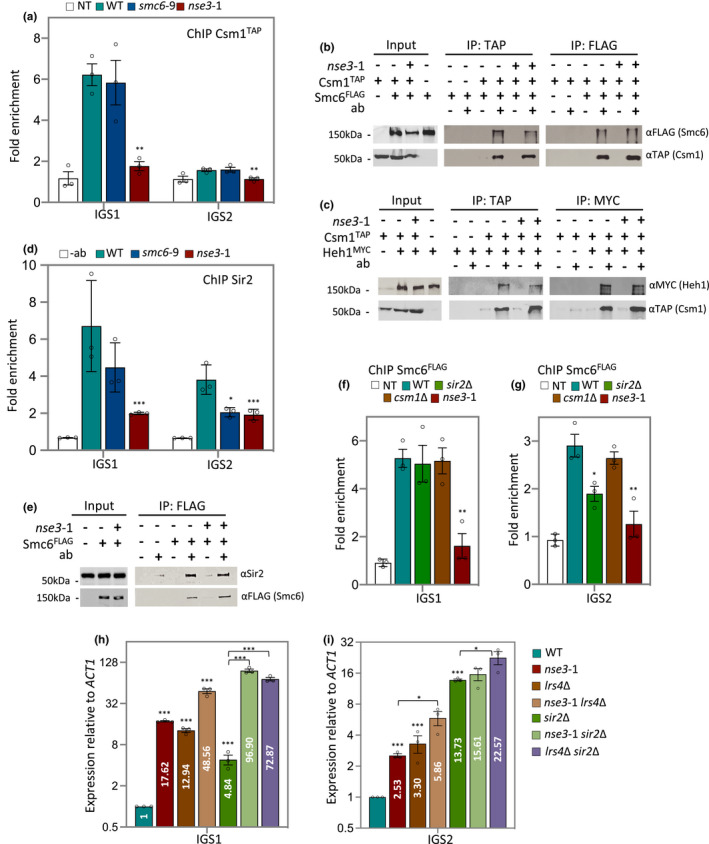
Interplay between Smc5/6, Cohibin and RENT maintain Transcriptional silencing at IGS1 and IGS2. (a) Enrichment of Csm1^TAP^ at IGS1 and IGS2 by ChIP with α‐TAP in WT (JC 4233), *smc6‐9* (JC 4938) and *nse3‐1* (JC 4251) at IGS1 and IGS2. Fold enrichment is based on normalization to negative control described in Figure 1. (b) Co‐IP between Smc6^FLAG^ and Csm1^TAP^ followed with western blotting using corresponding antibodies to epitope tags on each protein. IPs were performed in negative control (JC 1594; for α‐TAP IP) or (JC 4233; for α‐FLAG IP), WT (JC 4598) and *nse3‐1* (JC 4712). (c) Co‐IP between Csm1^TAP^ and Heh1^MYC^ followed with western blotting using corresponding antibodies to epitope tags on each protein. IPs were performed in negative control (JC 4224; for α‐TAP IP) or (JC 4233; for α‐MYC IP), WT (JC 4774) and *nse3‐1* (JC 4773). (d) Enrichment of Sir2 at IGS1 and IGS2 by ChIP with α‐Sir2 in WT (JC 471), *smc6‐9* (JC 1358) and *nse3‐1*(JC 3032) strains at IGS1 and IGS2. Fold enrichment is based on normalization to negative control region described in Figure 1 relative to no antibody control (beads only). (e) Co‐IP between Sir2 and Smc6^FLAG^ followed with western blotting using antibodies against Sir2 or FLAG. IP was performed in negative control (JC 471), WT (JC 1595) and *nse3‐1* (JC 3078). (f and g) Enrichment of Smc6^FLAG^ at IGS1 (f) and IGS2 (g) by ChIP with α‐FLAG in no‐tag control (NT; JC 471) WT (JC 1595), *sir2*Δ (JC 4699), *csm1*Δ (JC 4243) and *nse3‐1* (JC 3078). Fold enrichment is based on normalization to negative control region. (h and i)Transcription at IGS1 (h) and IGS2 (i) relative to WT cells after normalization to *ACT1* expression for WT (JC 471), *nse3‐1* (JC 3032), *lrs4*Δ (JC 3791), *nse3‐1* *lrs4*Δ (JC 3796), *sir2*Δ (JC 4648), *nse3‐1* *sir2*Δ (JC 3787) and *sir2*Δ *lrs4*Δ (JC 4979). Asterisks indicate statistical significance versus WT unless otherwise noted. Analysis was performed using at least three biological replicates. Statistical analysis is described in Section 4

We wanted to determine whether Smc5/6 impacted the localization of these complexes to the IGS regions. ChIP was performed with Csm1^TAP^ to measure Cohibin recovery in the rDNA. In *nse3‐1* cells there was a 3‐fold reduction in Csm1^TAP^ enrichment at IGS1 (Figure 3a). Consistent with previous report, at IGS2 the recovery of Csm1^TAP^ was very low compared to IGS1 (Mekhail et al., 2008) and it was statistically lower in *nse3‐1* compared to wild type (Figure 3a). By contrast, Csm1^TAP^ recovery at IGS1 and IGS2 in *smc6‐9* was indistinguishable from wild type (Figure 3a). In all, the recovery of Cohibin in the rDNA was partially dependent on Smc5/6, but independent of Smc5/6‐mediated HR processing. To determine whether *in* *vivo* physical interactions contributed to the interplay between Smc5/6 and Cohibin in the rDNA, a co‐IP was performed between Smc6^FLAG^ and Csm1^TAP^. Smc6^FLAG^ was recovered in α‐TAP (Csm1) pulldowns and vice versa, Csm1^TAP^ was recovered in α‐FLAG (Smc6) IPs (Figure 3b). Similar co‐IP experiments in *nse3‐1* mutant cells showed unchanged interactions between Smc5/6 and Cohibin (Figure 3b). Moreover, Y2H experiments showed Lrs4 and Csm1 interacted most strongly with Nse6 and Mms21, respectively (Figure S3a–e), prompting us to determine whether the interaction between Cohibin and the CLIP complex was mediated by Smc5/6 binding in the rDNA. Consistent with previous reports, we observed binding between Csm1^TAP^ and Heh^MYC^ by co‐IP (Figure 3c; Huang et al., 2006; Mekhail et al., 2008), and this was not altered in *nse3‐1* mutant cells (Figure 3c).

Taken together, these data show that Cohibin interacts with Heh1 independently of both Smc5/6 and Cohibin localization in the IGS regions.

We next measured Sir2 as a subunit of the RENT complex in the rDNA (Shou et al., 1999). In wild type cells, ChIP with α‐Sir2 showed ~6‐fold and 4‐fold enrichment above the non‐antibody control at IGS1 and IGS2, respectively (Figure 3d). In *nse3‐1* mutants, Sir2 enrichment at IGS1 was markedly reduced but still above control, whereas enrichment in *smc6‐9* was similar to wild type (Figure 3d). By contrast, Sir2 recovery at IGS2 in both *smc6‐9* and *nse3‐1* mutants was significantly reduced compared to wild type, but above the non‐antibody control (Figure 3d).

The interplay between Smc5/6 and RENT in the rDNA might be partially dependent on physical interactions as Sir2 was recovered in α‐FLAG (Smc6) pulldowns (Figure 3e). The reverse co‐IP experiment was not conducted due to limited antibody availability, however, as with Csm1, Sir2 association with Smc6 remained unaltered in *nse3‐1* mutants (Figure 3e). Taken together these data demonstrate that Smc5/6 binding at IGS1 and IGS2 is important for the localization of Cohibin and RENT, and that physical interactions between Smc5/6 and these complexes persist even when not recovered in the rDNA.

### 2.4 Smc5/6 functionality in the rDNA is independent of Cohibin and partially dependent on RENT

We next determined whether Cohibin or RENT contributed to the localization of Smc5/6 by performing ChIP on Smc6^FLAG^. ChIP with Smc6^FLAG^ substantiated the data obtained with Smc5^FLAG^, wherein the Smc5/6 complex is enriched at IGS1 and 2, and significantly reduced in *nse3‐1* mutant cells (Figure 3f,g). Smc6^FLAG^ recovery at IGS1 and IGS2 in *csm1*Δ was similar to wild type (Figure 3f,g). In contrast, Smc6^FLAG^ recovery at IGS1 was similar to wild type in *sir2*Δ mutants, however, recovery at IGS2 decreased to ~70% wild type levels (Figure 3g). These data suggest that the localization of Smc5/6 to IGS1 and IGS2 contributes to the overall stability of Cohibin and RENT more than the reverse, as only the deletion of *SIR2*, but not *CSM1*, impacted Smc5/6 association and only at IGS2 (Figure 3a,d,f and g).

In addition to the morphological changes (Figure 2a), transcriptional silencing is another pathway where Smc5/6, Cohibin and Sir2 might functionally converge (Huang et al., 2006; Mekhail et al., 2008; Unal et al., 2011; Zhao & Blobel, 2005). Thus, we investigated the interplay between Smc5/6 and these canonical factors in silencing at IGS1/2 in the rDNA (Bryk et al., 1997; Corbett et al., 2010; Fritze et al., 1997; Gottlieb & Esposito, 1989; Huang et al., 2006; Imai et al., 2000; Li et al., 2006; Mekhail et al., 2008; Rabitsch et al., 2003; Shou et al., 1999; Smith & Boeke, 1997; Smith et al., 1999; Straight et al., 1999; Vasiljeva et al., 2008; Visintin et al., 1999). At IGS1, transcription in *nse3‐1* (17.62) and *lrs4*Δ (12.94) was greater than in *sir2*Δ (4.84), but transcription synergistically increased in double mutants where *SIR2* was deleted, as in *nse3‐1* *sir2*Δ (96.90) and *lrs4*Δ *sir2*Δ (72.87; Figure 3h). A synergistic increase was also observed with *smc6‐9* *sir2*Δ (22.43; Figure S4a). Even though the level was lower than with *nse3‐1* *sir2*Δ, these data show that HR processing by Smc5/6 contributes to transcriptional regulation more in mutants with an underlying silencing defect.

At IGS2, the level of transcription in *sir2*Δ (13.73) was ~4‐fold higher compared to *nse3‐1* (2.53) and *lrs4*Δ (3.30; Figure 3i). Moreover, transcription in *nse3‐1* *sir2*Δ and *smc6‐9* *sir2*Δ was not markedly different than in *sir2*Δ single mutants (Figure 3i and Figure S4b), suggesting the lower level of Sir2 at IGS2 in *smc6‐9* and *nse3‐1* mutants did indeed contribute to silencing (Figure 3d).

In all, Sir2 and Cohibin recovery was higher at IGS1 than IGS2, and reduced at both regions in *nse3‐1* mutants (Figure 3a,d). Moreover, silencing defects were greater at IGS1 than IGS2, which likely stems from prolonged open chromatin and dynamic processing events at the E‐pro and RFB (Figure 3h,i and Figure S4a,b). Changes in transcription, together with the physical interactions existing between Smc5/6, Sir2, and Cohibin underscores the interdependent relationship of these silencing factors and their impact on rDNA chromatin structure and accessibility.

These factors converge at the rDNA and all have an interaction with the replication fork barrier protein, Fob1. Cohibin and Sir2 are recruited to the RFB in IGS1 by Fob1 and the accumulation of HR intermediates in Smc5/6 complex mutants is reversed by deleting *FOB1* (Buck et al., 2016; Huang et al., 2006; Menolfi et al., 2015; Peng et al., 2018; Torres‐Rosell, De Piccoli, et al., 2007; Torres‐Rosell et al., 2005). The recovery of Csm1 and Sir2 at IGS1 in *smc6‐9* was similar to wild type (Figure 3a,d), however, consistent with previous reports, their association decreased in *fob1*Δ mutants (Figure S4c,d; Buck et al., 2016; Huang et al., 2006; Huang & Moazed, 2003). Given the interplay of Smc5/6 with Cohibin and Sir2 and the differences in silencing at IGS1 and IGS2, we turned to investigate the function(s) of Smc5/6 in rDNA homeostasis in relation to Fob1.

### 2.5 Smc5/6 complex plays a Fob1‐independent role in modulating lifespan

**FIGURE 4:**
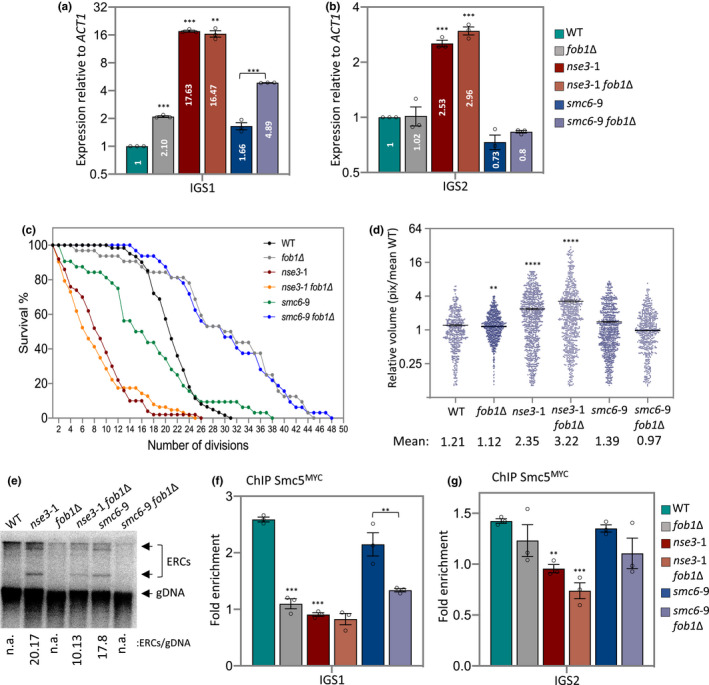
Smc5/6 function at IGS2 is important for nucleolar homeostasis independent of HR processing at the RFB. (a and b) Transcription of IGS1 (a) and IGS2 (b) measured and represented as relative to WT cells after normalization to *ACT1* expression for WT (JC 471), *fob1*Δ (JC 4825), *nse3‐1* (JC 3032), *nse3‐1* *fob1*Δ (JC 4595), *smc6‐9* (JC 1358) and *smc6‐9* *fob1*Δ (JC 4824) strains. (c) Replicative lifespan measured and represented as percentage of survival of mother cells with each division for WT (JC 471), *fob1*Δ (JC 4825), *nse3‐1* (JC 3032), *nse3‐1* *fob1*Δ (JC 4595), *smc6‐9* (JC 1358) and *smc6‐9* *fob1*Δ (JC 4824) strains. (d) Scatter plot data of nucleolar volume for WT (JC 5016), *fob1*Δ (JC 4985), *nse3‐1* (JC 5015), *nse3‐1* *fob1*Δ (JC 5110), *smc6‐9* (JC 5014) and *smc6‐9* *fob1*Δ (JC 5113) strains were measured in pixel and represented relative to mean of WT. (e) ERC molecules abundance in WT (JC 471), *fob1*Δ (JC 4825), *nse3‐1* (JC 3032), *nse3‐1* *fob1*Δ (JC 4595), *smc6‐9* (JC 1358) and *smc6‐9* *fob1*Δ (JC 4824) strains. (f and g) Enrichment of Smc5^MYC^ at IGS1 (f) and IGS2 (g) by ChIP with α‐MYC in WT (JC 3467), *fob1*Δ (JC 5041); *nse3‐1* (JC 3483), *nse3‐1* *fob1*Δ (JC 5044), *smc6‐9* (JC 5039) and *smc6‐9* *fob1*Δ (JC 5040). Fold enrichment is based on normalization to negative control region as described in Figure1. Asterisks indicate statistical significance versus WT unless otherwise noted. Analysis was performed using at least three biological replicates. Statistical analysis is described in Section 4

Consistent with previous reports, deletion of *FOB1* results in a minor increase in transcription at IGS1 and an extension in lifespan (Figure 4a,c; Buck et al., 2016). Transcription of IGS1 in *fob1*Δ mutants was ~36‐fold below *sir2*Δ *lrs4*Δ, which might reflect the low levels of Sir2 still bound at IGS1 in *fob1*Δ (Figure 4a and Figure S4c,e). This interpretation was further supported by transcription levels being markedly lower in *nse3‐1* *fob1*Δ (16.46) compared to *nse3‐1* *sir2*Δ *lrs4*Δ triple mutant cells (427.48), where the loss of silencing was synergistic (Figure 4a and Figure S4e).

*FOB1* deletion did not impact the silencing defects of either *smc6‐9* or *nse3‐1* at IGS2 (Figure 4b). The silencing defects in the Smc5/6 complex mutants correlated with increased nucleolar volume (Figure 2a,b), and consistently the nucleolar volume remained enlarged in *nse3‐1* independently of *FOB1* status (Figure 4d). IGS1 transcription in *smc6‐9* *fob1*Δ (4.89) increased relative to *smc6‐9* (1.66), however, the nucleolar volume remained compact in *smc6‐9* *fob1*Δ suggesting increased transcription from IGS1 alone does not correlate with increased morphological volume (Figures 2b and 4d). We do not know whether increased nucleolar volume would correlate with silencing defects in IGS2, independently of IGS1 as all mutants characterized here with increased transcription at IGS2 also showed increased transcription at IGS2 (Figure 4d).

Increased nucleolar morphology is linked to rDNA instability and lifespan. The reduced lifespan of *smc6‐9* was completely reversed by deletion of *FOB1*, and *smc6‐9* *fob*1Δ lived as long as *fob*1Δ mutants (Figure 4c). This is notable as deleting *FOB1* in *sir2*Δ mutants restored lifespan, but only to wild type (Kaeberlein et al., 1999). In stark contrast, the shortened lifespan of *nse3‐1* did not change in combination with *fob*1Δ (Figure 4c). These data highlight the importance of Smc5/6 in rDNA stability independently of HR‐mediated events at the RFB.

**FIGURE 5:**
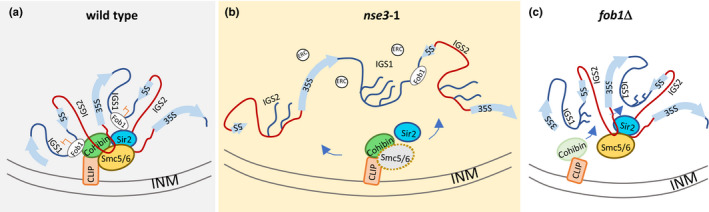
Schematic model for Smc5/6 functionality at the rDNA in the nucleolus. (a) In WT cells, nucleolar morphology is compact. Smc5/6 binds to the rDNA array at IGS1 and IGS2 and physically interacts with chromatin and canonical rDNA factors, Sir2, Cohibin. IGS regions are silenced and the repeats are tethered to the periphery through interaction with the CLIP complex. (b) In *nse3‐1* mutant cells, Smc5/6 fails to bind rDNA repeats, yet it still physically interacts with Sir2, Cohibin and Heh1. Loss of the Smc5/6 complex results in defective silencing at both IGS1 and IGS2, accumulation of ERC molecules and increased nucleolar volume. (c) In *fob1*Δ mutants, the binding of Sir2, Cohibin and Smc5/6 with IGS1 is reduced and transcription from IGS1 increases. Tethering through CLIP is lost, however, all factors bind and silence at IGS2 and the nucleolar morphology is compact

One measure of rDNA instability is the production of ERCs that arise from recombination intermediates (Ganley & Kobayashi, 2014; Sinclair & Guarente, 1997; Takeuchi et al., 2003). Fob1 binding at the RFB is central in this process and Smc5/6 is likely to be involved as it modulates HR processing at stalled replication forks (Buck et al., 2016; Huang & Moazed, 2003; Johzuka & Horiuchi, 2002; Kobayashi, 2003; Kobayashi & Horiuchi, 1996; Peng et al., 2018; Torres‐Rosell, De Piccoli, et al., 2007; Torres‐Rosell, Sunjevaric, et al., 2007). Indeed, ERC levels increased in both *smc6‐9* and *nse3‐1* mutant cells (Figure 5e and Figure S5a,b). Consistent with previous work the production of ERCs decreased in *fob*1Δ because forks no longer stall at the RFB (Defossez et al., 1999; Johzuka & Horiuchi, 2002). ERC formation in *smc6‐9* depended on *FOB1*+, which correlated with the lifespan extension. However, in *nse3‐1* mutants, the level of ERCs reduced but were still detectable in *nse3‐1* *fob1*Δ (Figure 4e). These data support the model that ERC levels coincide with, but do not cause lifespan reduction, as this was similar in *nse3‐1* and *nse3‐1* *fob*1Δ mutants. Moreover, these data indicate that *fob1*Δ is able to rescue Smc5/6 HR‐defects, but not Smc5/6 localization defects. Thus, the importance of the complex in rDNA stability is not entirely dependent on Fob1 binding at the RFB (Figure 4e and Figure S5a–e). These Fob1‐independent functions might be linked to Smc5/6 at IGS2 as the level of Smc5^MYC^ recovered at IGS1, but not IGS2, reduced in cells where *FOB1* was deleted (Figure 4f,g). Fob1 binding of IGS1 was not altered in cells carrying either *nse3‐1* or *smc6‐9* (Figure S5f). Taken together, these data support a role for Smc5/6 in transcriptional silencing and rDNA repeat compaction involving binding of the complex at IGS2 independently of Fob1 (Figure 5a).

## 3 DISCUSSION

Here we demonstrate a previously uncharacterized function for Smc5/6 within the rDNA involving two interrelated mechanisms. One in transcriptional silencing at IGS1 and IGS2 and the other in chromosome organization and sequestration of the repeats at the periphery. We show that Smc5/6 physically and genetically interacts with Sir2 and Cohibin (Lrs4/Csm1). Similar to these canonical silencing factors, Smc5/6 binds in the IGS regions and also interacts with Heh1 of the CLIP complex, a INM factor important for tethering the rDNA repeats at the nuclear periphery (Figure 5a; Chan et al., 2011; Kaeberlein et al., 1999). The binding of all these factors at IGS1/2 was markedly reduced when Smc5/6 did not localize to the rDNA in *nse3‐1* mutants (Figure 5b). While these complexes are also known to bind other genomic loci including telomeres, centromeres and the mating type loci, the impact on lifespan likely stems from events in the rDNA as there is no correlation between telomere length and replicative lifespan in budding yeast (Austriaco & Guarente, 1997; Harari et al., 2017; Peng et al., 2015).

The association of Smc5/6 with Sir2 and Cohibin was unaltered in *nse3‐1* mutants, thus their association elsewhere might persists (Chan et al., 2011; Corbett et al., 2010; Gottschling et al., 1990; Huang et al., 2006; Ivy et al., 1986; Lindroos et al., 2006; Mekhail et al., 2008; Menolfi et al., 2015; Moradi‐Fard et al., 2016; Rine & Herskowitz, 1987; Torres‐Rosell et al., 2005; Tsukamoto et al., 1997). The differences in the rDNA we observed by comparing *nse*3‐1 and *smc5*‐6 showed that while both IGS regions are linked to repeat stability and lifespan, IGS1 maintenance involves Fob1‐dependent HR processing whereas IGS2 involves Sir2 and Smc5/6 binding, independently of Fob1. Silencing and nucleolar compartmentalization was minimally impacted in HR‐deficient Smc5/6 which localized efficiently to IGS1 (Peng et al., 2018; Torres‐Rosell, De Piccoli, et al., 2007; Torres‐Rosell, Sunjevaric, et al., 2007). Moreover, cells harboring *smc6‐9* showed smaller transcriptional changes compared to *nse3‐1*, but increases were observed compared to wild type. Our data support a model that HR defects at IGS1, rather than silencing defects, led to the reduced lifespan we observed in *smc6‐9* mutants. Deletion of *FOB1* reversed ERCs and the lifespan defects in *smc6*9 mutants, however silencing defects persisted, even increased at IGS1 (Figure 5c). This was true for *fob1*Δ *and* *smc6‐9* *fob1*Δ mutants and is consistent with previous work showing that Fob1‐dependent fork pausing and transcriptional silencing at IGS1 are separately regulated (Bairwa et al., 2010).

The CLIP complex binds IGS1 and IGS2, and its association depends on Smc5/6 and Fob1. (Figure 2c and Figure S6). The reduced lifespan of *heh1*Δ mutants was previously shown to be reversed by *FOB1* deletion (Chan et al., 2011) However, in contrast to *nse3‐1* mutants, the compaction of the rDNA, as measured by increased transcription and morphological expansion of the nucleolus, did not depend on Heh1 binding in the IGS regions as both *heh1*Δ and *fob1*Δ mutants maintained a compact nucleolus (Figures 2b and 4d; Chan et al., 2011). In all, these data argue that decreased chromatin organization and silencing in IGS2, rather than decreased tethering via CLIP manifests as nucleolar expansion.

A number of studies have linked abnormalities in nucleolar morphology with premature aging and naturally aged cells (Matos‐Perdomo & Machin, 2019; Mehta et al., 2007; Sinclair et al., 1997). For instance, the enlarged nucleolus of old cells become more compact when lifespan is extended upon induction of a ‘rejuvenation factor’ in old cells (Unal et al., 2011). Increased morphology might stem from defects in chromatin organization driven by reduced Sir2 or Smc5/6 binding at IGS2. This is supported by silencing defects at IGS2 in mutants with shortened lifespans, as shown here for *nse3‐1* and previously for *sir2*Δ and *lrs4*Δ (Bryk et al., 1997; Corbett et al., 2010; Fritze et al., 1997; Huang et al., 2006; Mekhail et al., 2008; Smith & Boeke, 1997). Sir2 and Cohibin also bind and silence IGS1 (Figure 3h). However, their binding levels at IGS1 do not appear to regulate lifespan as their recovery was reduced in both *smc6‐9* *fob1*Δ and *nse3‐1* *fob1*Δ mutants which have a lifespan extension and reduction, respectively (Figure 4c and Figure S4c,d; Bairwa et al., 2010; Huang et al., 2006). Our data suggest that increased morphology correlates with silencing defects at IGS2, which is high in *nse3‐1* and *nse3‐1* *fob1*Δ, but not in *smc6‐9* *fob1*Δ double mutants.

In conclusion, we show that the loss of Smc5/6 binding in the rDNA correlates with a loss of nucleolar compaction, a loss of transcriptional silencing at IGS2 and a reduced lifespan. These functions are independent from canonical HR‐mediated roles of Smc5/6 complex at rDNA and not reversed by the deletion of *FOB1*. RNA polymerase I is essential for Sir2 binding to IGS2 and rDNA silencing (Buck et al., 2002; Huang & Moazed, 2003). Therefore, investigating the interplay between RNA Pol I, Sir2 and Smc5/6 could address the relationship between IGS2‐based silencing, rDNA structural compaction and replicative lifespan.

## 4 EXPERIMENTAL PROCEDURES

All the yeast strains used in this study are listed in Table S1 and were obtained by crosses. The strains were grown on various media for the experiments and are described below. For all experiments filter sterilized YPAD (1% yeast extract, 2% bactopeptone, 0.0025% adenine, 2% glucose and 2% agar) media were used. For yeast 2‐hybrid assays, standard amino acid drop‐out media lacking histidine, tryptophan and uracil were used and 2% raffinose was added as the carbon source for the cells. In all experiments, exponentially growing cells were incubated at 30°C for 2 h before harvesting, unless indicated otherwise.

### 4.1 Chromatin immunoprecipitation

ChIP experiments performed as described previously (Tittel‐Elmer et al., 2009). Cells were grown over night at 25°C, then diluted to 1 × 10^7^ cells/ml in liquid YPAD and incubated at 30°C for 2 h before crosslinking with 1% formaldehyde (Sigma) for 15 min followed by quenching with 125 mM glycine for 5 min at room temperature. Fixed cells were washed three times with cold PBST (phosphate buffered saline with Tween 20) and froze over night at −80°C. Cells were lysed in lysis buffer (50 mm HEPES, 140 mm NaCl, 1 mm EDTA, 1% Triton X‐100, 1 mM PMSF and protease inhibitor pellet), the clarified by spinning at 20,000 *g* for 15 min (at 4°C). Pellets were sonicated for 12 × 15 s at amplitude of 50% with 45 s shut off intervals and immunoprecipitated using corresponding antibodies. Precipitates were washed once with lysis buffer and twice with wash buffer (100 mM Tris (pH 8), 0.5% Nonidet P‐40, 1 mM EDTA, 500 mM NaCl, 250 mM LiCl, 1 mM PMSF and protease inhibitor pellet (Roche)) at 4°C, each for 5 min shaking at 2,200 *g*. Real‐time qPCR reactions were carried on using power up SYBR green master mix on a QuantStudio™ 6 Flex Real‐Time PCR System (Applied Biosystems, Life Technologies Inc.). Ct (cycle threshold) values of Ab‐coupled beads and uncoupled beads used to calculate fold enrichment of protein on rDNA regions relative to an unrelated genomic locus ZN (for ChIP experiments), or *ACT1* (for expression at rDNA).

### 4.2 Co‐immunoprecipitation

Strains were grown overnight at 25°C and then diluted and grown to the log phase by incubating for 2 h at 30°C in YPAD media. Cells were lysed with zirconia beads in lysis buffer (50 mm HEPES, 140 mm NaCl, 1 mm EDTA, 1% Triton X‐100, 1 mM PMSF and protease inhibitor pellet). Cell lysates were incubated with antibody‐coupled Dynabeads for 2 h at 4°C. Immunoprecipitates were washed end over end once with lysis buffer and twice with wash buffer (100 mM Tris (pH 8), 0.5% Nonidet P‐40, 1 mM EDTA, 250 mM LiCl, 1 mM PMSF and protease inhibitor pellet), each for 5 min. Beads were resuspended in SDS loading buffer and subjected to SDS gel electrophoresis followed by western blotting using appropriate antibodies listed in the resource table.

### 4.3 qPCR based gene expression analyses

Cells were grown over night at 25°C, then diluted to 5 × 10^6^ cells/ml in liquid YPAD and incubated at 30°C for 2 h before fixing the cells with 1% Sodium azide. Fixed cells were washed with cold PBS (phosphate buffered saline; 1.37 M NaCl, 27 mM KCl, 100 mM Na_2_HPO_4_, 18 mM KH_2_PO_4_) and snap frozen in liquid nitrogen. Next day, cells were lysed using RNeasy kit reagents and isolated RNA was subjected to reverse transcription. Complementary DNA (cDNA) was amplified and quantified using the SYBR Green qPCR method. Primers are listed in Table S2. Expression values represent real time qPCR values relative to *ACT1* and normalization to WT samples.

### 4.4 PFGE

Saturated overnight culture cells were diluted to 1 × 10^7^ cells/ml in liquid YPAD and incubated at 30°C for 2 h. Cell cultures were adjusted to 1 × 10^7^ cells/ml; 50 ml. Cells were killed in 0.1% Sodium azide and washed with cold TE^50^ (10 mM Tris–HCl, pH 7.0, 50 mM EDTA, pH 8.0). To avoid mechanical shearing of genomic DNA, cells were solidified in 1% low melting‐point CHEF‐quality agarose in plug moulds (5 × 10^7^ cells/plug) at 4°C. Plugs were incubated overnight in 0.1 M sodium phosphate pH 7.0, 0.2 M EDTA, 40 mM DTT, 0.4 mg/ml Zymolyase 20T at 37°C, washed few times with TE^50^ and incubated in 0.5 M EDTA, 10 mM Tris–HCl pH 7.5, 1% *N*‐lauroyl sarcosine, 2 mg/ml proteinase K for 48 h at 37°C. Plugs were then washed with cold TE^50^ and stored at 4°C until subjected to electrophoresis. Chromosomes were separated on a CHEF‐DRII instrument (Bio‐Rad) for 68 h at 3.0 V/cm, 300–900 s, 14°C on a 0.8% CHEF agarose gel in 0.5% TBE. EtBr‐stained gels were destained and then subjected to standard southern blotting as previously described (Moradi‐Fard et al., 2016). Briefly, gels were treated with 0.25 N HCl for 20 min then in 0.5 M NaOH, 3 M NaCl for 30 min for in‐gel depurinating and denaturing of genomic DNA, respectively. Denatured DNA were transferred to Amersham Hybond‐XL membrane overnight. Membranes were then crosslinked by UV Stratalinker 1800 (120 mJoules) and hybridized with radio‐labeled rDNA specific probe (Unal et al., 2011). Rediprime II DNA Labeling System used to radiolabel rDNA probe.

### 4.5 Visualization of ERC molecules

Genomic DNA were prepared using standard protocol or made in plugs (as described in PFGE section). ~2 µg of gDNA or plugs used to run in 0.7% agarose gel; 0.5× TBE. DNA fragments were separated for ~24 h at 40 V, 4°C. Gels were then subjected to standard southern blotting and probed with rDNA‐specific probe as described in PFGE section. ERC molecules were measured and represented after normalizing to genomic rDNA band using the ImageJ software.

### 4.6 Microscopy

Cells were grown overnight at 25°C and diluted to 5 × 10^6^ cells/ml and grown at 30°C to reach a concentration of 1 × 10^7^ cells/ml. Cells were washed twice with SK buffer (0.05 M KH_2_PO_4_, 0.05 M K_2_HPO_4_, 1.2 M Sorbitol). And mounted on slide for imaging. 15 Z‐stack images were obtained with 0.3 µm increments along the *z*‐plane to cover a total range of cells nuclei at 60× magnification and 1.5 µm/pixel zoom factor.

Three dimensional (*X*, *Y*, *Z*) stacks of yeast cells carrying Nop1‐CFP and/or Nup49‐GFP were acquired using the “Nikon Ti Eclipse Widefield” microscope provided by Live Cell Imaging facility at University of Calgary; ~200 and 400 ms exposure times used for GFP and CFP channels, respectively. The acquired 3D stacks were first deconvolved using Huygens software. 3D segmentation was done by thresholding (using the auto thresholding range recommended) in the ImageJ software using 3D manager plugin. The volume measurements were acquired in pixel and presented as relative to the obtained average volume (in pixel) for WT cells.

### 4.7 Replicative lifespan

Replicative lifespan assays were done as described (Postnikoff & Harkness, 2014). Cells from logarithmically growing liquid cultures were streaked on YPD plates. After an overnight incubation at 30°C, a minimum starting population of 32 newly budded cells were removed to start the experiment using a Zeiss Micro‐manipulator, where the new buds served as the virgin mother cells. Budded cells that harbored the *nse3*‐*1* allele showed low viability once selected from the initial streak, so many more cells were selected and followed to ensure an appropriate RLS was measured (*nse3*‐*1*, *n* = 50; *nse3*‐*1* *fob1*Δ, *n* = 63). Buds were successively dissected away and discarded until all mother cells had ceased dividing. The plates were maintained at 30°C while picking and stored at 4°C overnight.

### 4.8 Yeast 2‐hybrid

Various plasmids (Table S3) were constructed containing the gene encoding the proteins – Smc5, Nse1, Mms21, Nse3, Nse4, Nse6, Csm1, Lrs4 and Heh1 – using the primers listed in Table S2. The plasmids J 965 and J 1493 and the inserts were treated with corresponding enzymes and ligated using T4 DNA ligase. The plasmids were sequence verified. Reporter (J 359), bait (J 965) and prey (J 1493) plasmids, containing the gene encoding the desired protein, were transformed into JC 1280. Cells were grown overnight in media lacking uracil, histidine and tryptophan with 2% raffinose. Next day, cells were transferred into media lacking uracil, histidine and tryptophan with either 2% glucose or 2% galactose and grown for 6 h at 30°C. Cell pellets were resuspended and then permeabilized using 0.1% SDS followed by ONPG addition. βgalactosidase activity was estimated by measuring the OD at 420 nm, relative βgalactosidase units were determined by normalizing to total cell density at OD600.

### 4.9 Western Blot

Cells were lysed by re‐suspending them in lysis buffer (with PMSF and protease inhibitor cocktail tablets) followed by bead beating with zirconia beads. The protein concentration of the whole cell extract was determined using the Nanodrop (Thermo Scientific). Equal amounts of whole cell extract were added to SDS PAGE gel wells. Standard SDS PAGE protocol were performed. Proteins were then transferred to nitrocellulose membrane and detected using corresponding antibodies listed in the resource table.

### 4.10 Quantification and statistical analysis

Data in bar graphs represent the average of at least three biological replicates. Error bars represent the standard error of mean (SEM). Significance (*p* value) was determined using 1‐tailed, unpaired Student's *t* test – *p < 0.05; **p < 0.01; ***p < 0.001. Statistical analyses were performed in Prism version 7 (GraphPad). Kruskal–Wallis test was performed to determine statistical significance between nucleolar volumes measured for indicated strains – *p < 0.05; **p < 0.01; ***p < 0.001, ****p < 0.0001.

## CONFLICT OF INTEREST

The authors declare that they have no conflicts of interest with the contents of this article.

## AUTHORS CONTRIBUTIONS

S.M‐F., A.M., M.C., and T.A.A.H. performed experiments and analyzed the data. J.A.C, S.M‐F, and A.M. designed experiments and wrote the manuscript.

## Supporting information

Supplementary Material

## Data Availability

This study did not generate/analyze any code. Original data supporting the figures in the paper is available from the corresponding author on request.
