# Supplementary material for: Smc5/6 in the rDNA modulates lifespan independently of Fob1​
Source: Aging Cell. 2021 May 12;20(6):e13373. doi: 10.1111/acel.13373 (PMC8208791; doi:10.1111/acel.13373)
Supplement: Supplementary file 1 — Supplementary Material [file ACEL-20-e13373-s001.pdf]

SUPPLEMENTAL INFORMATION

Supplemental information includes 3 tables and 6 figures and can be found with this article. Yeast strains used in this study are listed in Table S1. Details of plasmids and primers used in this study are specified in Table S2 and S3.

Table S1: Strains used is this study

Strain	***Genotype***	***Source***
JC470	*MAT***a** *ade2-1 trp1-1 his3-11 his3-15 ura3-1 leu2-3 leu2-112 Rad5+ (W303)*	R. Rothstein
JC471	*MATα ade2-1 trp1-1 his3-11 his3-15 ura3-1 leu2-3 leu2-112 Rad5+ (W303)*	R. Rothstein
JC1280	*MATα his3 trp1 ura3-52 leu2::proLEU2-lexAop6*	Golemis, et al, 1996
JC1358	JC471 with *smc6*-9::*KanMX6*	C. Boone Lab
JC1594	JC470 with Smc6-6HIS-3FLAG::*KanMX4*	Bustard et al., 2012
JC1595	JC471 with Smc6-6HIS-3FLAG::*KanMX4*	this study
JC3032	JC471 with *nse3*-1::*HYG*	Moradi-Fard et al., 2016
JC3039	JC470 with *smc6*-9::*KanMX6*	this study
JC3078	JC1595 with *nse3*-1::*URA3*	this study
JC3084	JC470 with *nse3*-1::*URA3*	this study
JC3467	JC470 with Smc5-13MYC::*KanMX6*	this study
JC3483	JC3467 with *nse3*-1::*URA3*	this study
JC 3728	JC470 with Smc5-6HIS-3FLAG::*KanMX4*	this study
JC3787	JC470 with *sir2*::*TRP1*, *nse3*-1::*HYG*	this study
JC3790	JC471 with *lrs4*::*KanMX6*	this study
JC3791	JC470 with *lrs4*::*KanMX6*	K. Mekhail lab
JC3796	JC3791 with *nse3*-1::*HYG*	this study
JC4022	JC470 with Heh1-13MYC::*KanMX6*	this study
JC4107	JC470 with Heh1-TAP::*TRP1*, TELVR::*ADE2*	K. Mekhail lab
JC4205	JC1595 with *heh1*::*HYG*	this study
JC4224	JC471 with Heh1-13MYC::*KanMX6*	this study
JC4228	JC4022 with *nse3*-1::*HYG*	this study
JC4233	JC471 with Csm1-TAP::*TRP1*	K. Mekhail lab
JC4243	JC1595 with *csm1*::*KanMX6*	this study
JC4251	JC4233 with *nse3*-1::*HYG*	this study
JC4595	JC3032 with *fob1::HIS3*	this study
JC4598	JC1594 with Csm1-TAP::*TRP1*	this study
JC4648	JC471 with *sir2*::*TRP1*	this study
JC4676	JC471 with Nup49-*GFP*, Nop1-CFP::*URA3*	this study
JC4699	JC1595 with *sir2*::*TRP1*	this study
JC4712	JC4598 with *nse3*-1::*HYG*	this study
JC4729	JC470 with Nup49-*GFP*, Nop1-CFP::*URA3*, *nse3*-1::*HYG*	this study
JC4731	JC4676 with *lrs4*::*KanMX6*	this study
JC4733	JC4676 with *sir2*::*TRP1*	this study
JC4735	JC4676 with *heh1*::*HYG*	this study
JC4773	JC4774 with *nse3*-1::*HYG*	this study
JC4774	JC4224 with Csm1-TAP::*TRP1*	this study
JC4811	JC1595 with Heh1-TAP::*TRP1*	this study
JC4813	JC4811 with *nse3*-1::*URA3*	this study
JC4823	JC3039 with *fob1::LEU2*	this study
JC4824	JC1358 with *fob1::LEU2*	this study
JC4825	JC471 with *fob1::LEU2*	this study
JC4929	JC4824 *with* Csm1-TAP::*TRP1*	this study
JC4932	JC4676 with *smc6*-9::*KanMX6*	this study
JC4937	JC4233 with *fob1::LEU2*	this study

JC4938	JC4233 with *smc6*-9::*KanMX6*	this study
JC4940	JC4022 with *fob1::LEU2*	this study
JC4942	JC4022 with *smc6*-9::*KanMX6*	this study
JC4943	JC4022 with *smc6*-9::*KanMX6*, *fob1::LEU2*	this study
JC4973	JC4233 with *fob1::LEU2*, *nse3*-1::*HYG*	this study
JC4975	JC4022 with *fob1::LEU2*, *nse3*-1::*HYG*	this study
JC4976	JC1358 with *lrs4*::*KanMX6*	this study
JC4978	JC1358 with *sir2*::*HIS3*	this study
JC4979	JC3790 with *sir2*::*HIS3*	this study
JC4980	JC4979 with *nse3*-1::*HYG*	this study
JC4985	JC5016 with *fob1::LEU2*	this study
JC5007	JC471 with Fob1-3HA:: *HIS3*	this study
JC5008	JC5007 with *nse3*-1::*HYG*	this study
JC5010	JC5007 with *smc6*-9::*KanMX6*	this study
JC5014	JC5016 with *smc6*-9::*KanMX6*	this study
JC5015	JC5016 with *nse3*-1::*HYG*	this study
JC5016	JC471 with Nop1-CFP::*URA3*	this study
JC5017	JC5016 with *sir2*::*TRP1*	this study
JC5018	JC5016 with *heh1*::*HYG*	this study
JC5019	JC5016 with *lrs4*::*KanMX6*	this study
JC5039	JC3467 with *smc6*-9::*KanMX6*	this study
JC5040	JC5039 with *fob1::LEU2*	this study
JC5041	JC3467 with *fob1::LEU2*	this study
JC5044	JC3483 with *fob1::LEU2*	this study
JC5110	JC5015 with *fob1::HIS3*	this study
JC5113	JC5014 with *fob1::HIS3*	this study
JC5879	JC3728 with *nse3*-1::*HYG*	this study
JC5882	JC3032 with *fob1::HIS3*	this study
JC5883	JC470 with *nse3*-1::*HYG*, *fob1::HIS3*	this study
JC5894	JC3728 with *smc6*-9::*KanMX6*	this study

Table S2: Oligos used in this study

Primer name	***Primer number***	***Sequence 5'> 3'***
NTS1	C1577	AGGGCTTTCACAAAGCTTCC
	C1578	TCCCCACTGTTCACTGTTCA
NTS2	C1795	CCACCACACTCCTACCAATAAC
	C1796	AGGTAGTCAGATGAAAGATGAATAGAC
P1	C1791	CACACTATCATCCTCATCGTATATT
	C1792	AGAGAGAAGTAGACTGAACAAGT
P2	C1799	ACGATGAGAGACTGTTCAGGTTAAA
	C1800	GGGTTGATGCGTATTGAGAGATA
P3	C1801	CCAATTGTTCCTCGTTAAGGTATTT
	C1802	ATTCAGGGAGGTAGTGACAATAAA
P4	C1807	GTTTGAGAATAGGTCAAGGTCATTTC
	C1808	GTTTCCCTCAGGATAGCAGAAG
ZN	C1275	GCACTTAATTGGCGTAAGCTG
	C1276	TCGCAGGAGCATATTTCGTA
Act1	C1561	TGTCCTTGTACTCTTCCGGT
	C1562	CCGGCCAAATCGATTCTCAA
Smc5	C1483	GATCCCATGGATGACCAGTCTAATAGATTTGGGCAGATATG
	C1484	GATCCTCGAGTTAATCGAATGAGTAGTTAGAAGTTTCACCG
Nse1	C719	CTAGGAATTCATGGAGGTACATGAAGAGC
	C720	CTAGCTCGAGTTAAATAACGTATACGCCCTCTG
Nse3	C723	CTAGGAATTCATGAGTTCTATAGATAATGAC
	C724	CTAGCTCGAGCTATATAGAATATGAATCGCC
Nse4	C721	CTAGGAATTCATGTCTAGTACAGTAATATC
	C722	CTAGCTCGAGTAAGAATGGTGAAGTGATGTTG
Nse6 (pJ1493)	C609	GATCGGATCCGTGTCACAAATGGGAAGCGTGAACTCATCACCG
	C610	GATCCTCGAGCAGATCAATGTTCAGTCATCATGACTGTTACC TG
Nse6 (pJ965)	C892	GATCGGATCCAAATGGGAAGCGTGAACTCATCACCG
	C610	GATCCTCGAGCAGATCAATGTTCAGTCATCATGACTGTTACC TG
Csm1	C1737	GATC GAATTC ATGGATCCATTGACTGTATACAAAAACTCAGTGAAACA
	C1738	GATC CTCGAG TTATGTAGCAGCTTACTCGGTTTCATCTTTTTTCTCTC
Lrs4	C1735	GATCGAATTCATGGAGCATGTAGATTCCGATTTTGCACCTATAAGGAG
	C1736	GATC CTCGAG GATAGCTGTTACTCATACAAACTCGTCAACATTTAAAT
Heh1	C1898	GATCGCGGCCGCATGAATAGTGACTTGGAGTATTTAGAGGACGGTTTTGA
	C1915	GATCCTCGAGTCATTTTGTGGGTTATATTTTGTTTTCAGCGGAATCCT

Table S3: Plasmids Used in this study

Plasmid number	***Plasmid description***	***Source***
J 965	pGAL-lexA	S. Gasser lab
J 1493	pJG4-6	S. Gasser lab
J 359	pSH18-34 lexAGal1-lacZ	
J 1805	J 965 with Nse1	
J 102	J 965 with Mms21	
J 138	J 965 with Nse3	
J 1804	J 965 with Nse4	
J 038	J 965 with Nse5	
J 141	J 965 with Nse6	
J1902	J 965 with Smc5	
J 1874	J 965 with Csm1	
J 1875	J 965 with Lrs4	
J 048	J 1493 with Smc5	
J 063	J 1493 with Nse6	
J 050	J 1493 with Mms21	
J 1881	J 1493 with Heh1	
J 187	pFN4 pNOP1-CFP	1742 S. Gasser lab
J 1830	pNOY373, containing 9.1 Kb rDNA repeats	(Unal et al., 2011)

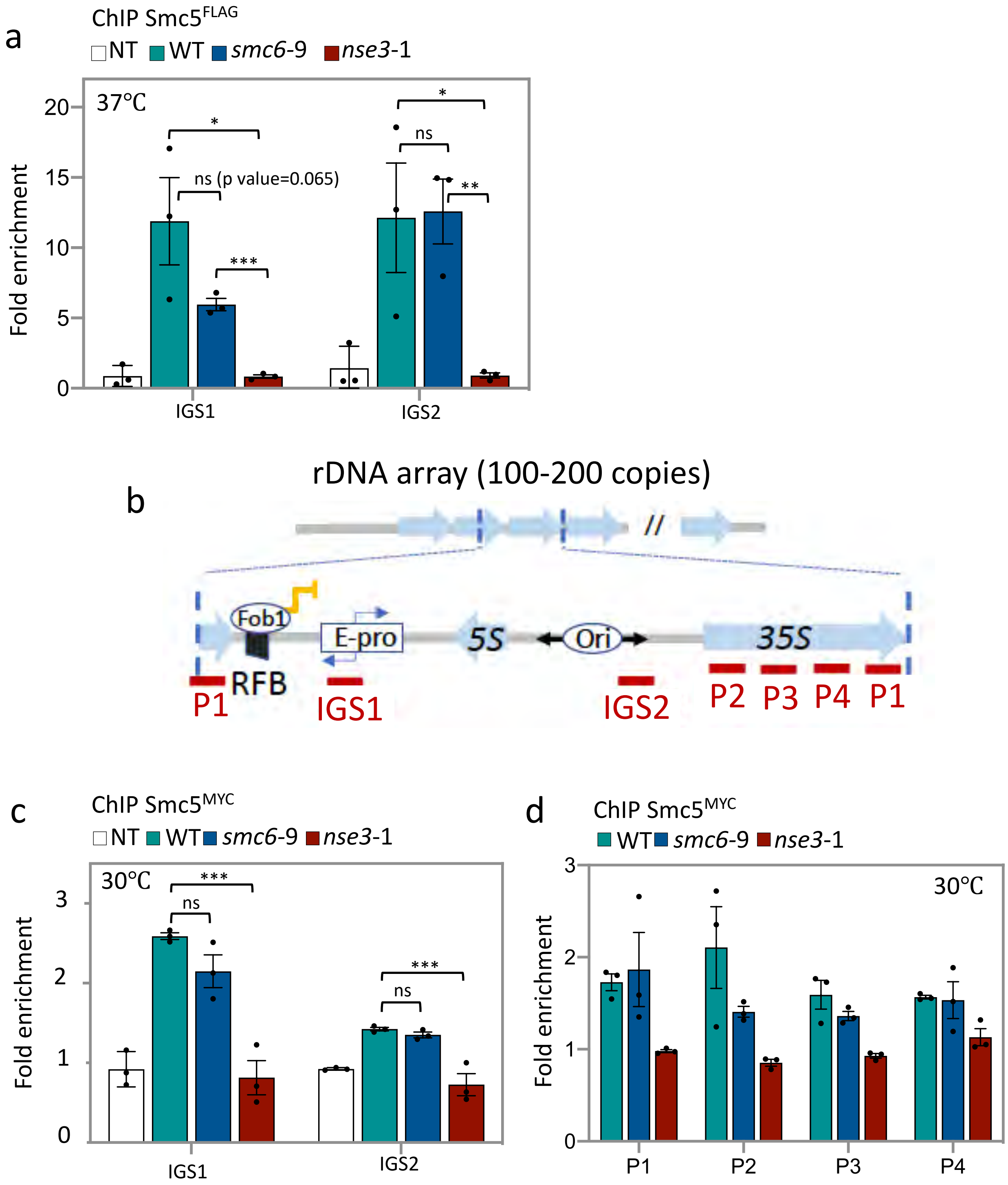

Supplementary Figure 1, Related to Figure 1.

a. Enrichment of Smc5^{MYC} at IGS1 and IGS2 with α-FLAG at 37℃ in non-tagged control (JC 470), WT (JC 3728), *nse3*-1 (JC 5879) and *smc6*-9 (JC 5894). Fold enrichment is based on normalization to negative control region as described in Fig.1. Analysis was performed using at least three biological replicates. Statistical analysis is described in methods.

b. Location of P1-P4 probes in the rDNA.

c., d. Enrichment of Smc5^{Myc} at IGS1 and IGS2 (b) or P1-P4 (c) by ChIP with α-MYC at 30℃ in non-tagged control (JC 470), WT (JC 3467), *nse3*-1 (JC 3483) and *smc6*-9 (JC 5039).

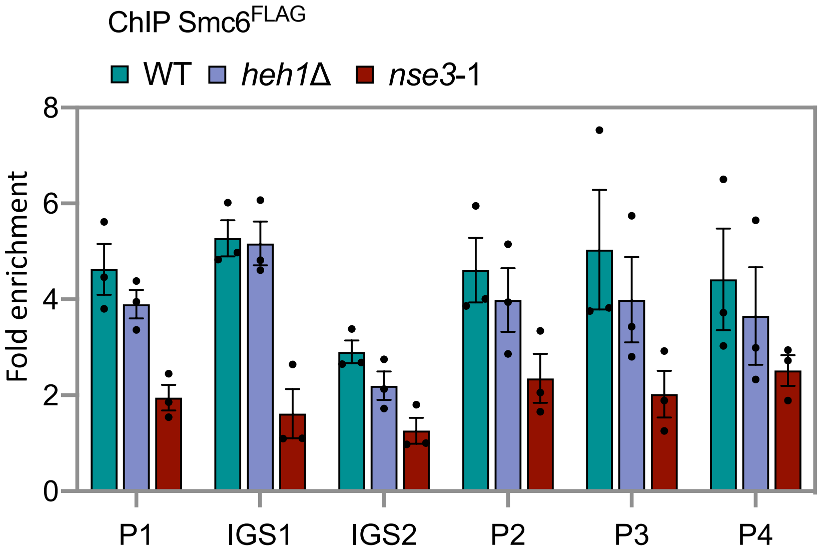

Supplementary Figure 2, Related to Figure 2.
Enrichment of Smc6^{FLAG} at IGS1 and IGS2 by ChIP with α-FLAG in WT (JC 1595), *heh1*Δ (JC 4205) and *nse3*-1 (JC 3078). Fold enrichment is based on normalization to negative control region as described in Fig.1. Analysis was performed using at least three biological replicates. Statistical analysis is described in methods.

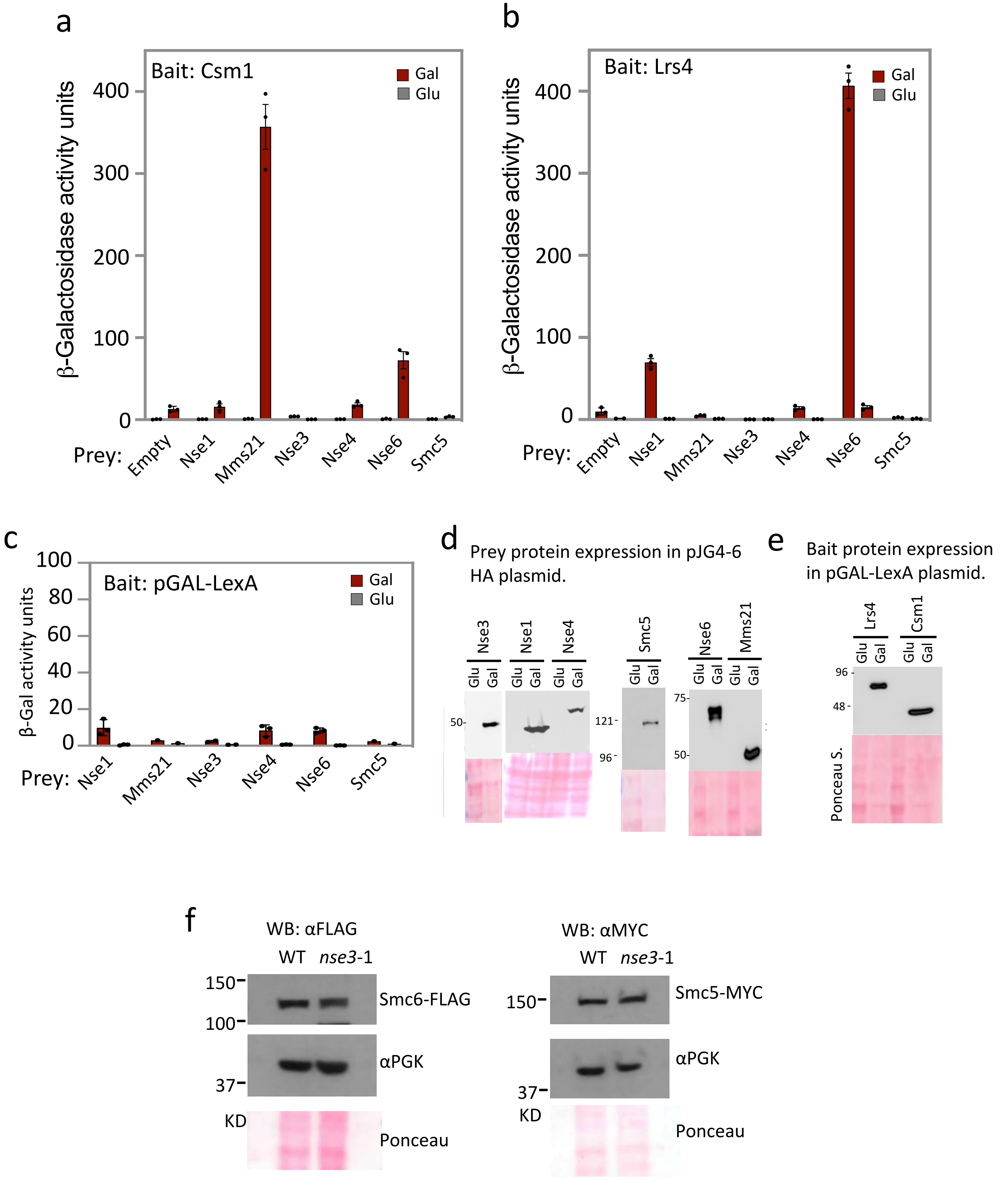
a
Bait: Csm1
Gal
Glu
β-Galactosidase activity units
400
300
200
100
0
Prey:
Empty
Nse1
Mms21
Nse3
Nse4
Nse6
Smc5
b
Bait: Lrs4
Gal
Glu
β-Galactosidase activity units
400
300
200
100
0
Prey:
Empty
Nse1
Mms21
Nse3
Nse4
Nse6
Smc5
c
Bait: pGAL-LexA
Gal
Glu
β-Gal activity units
100
80
60
40
20
0
Prey:
Nse1
Mms21
Nse3
Nse4
Nse6
Smc5
d
Prey protein expression in pJG4-6 HA plasmid.
Nse3
Nse1
Nse4
Smc5
Nse6
Mms21
Glu
Gal
50-
121-
96-
75-
50-
e
Bait protein expression in pGAL-LexA plasmid.
Lrs4
Csm1
Glu
Gal
96
48
Ponceau S.
f
WB: αFLAG
WT
nse3-1
150
100
37
KD
Smc6-FLAG
αPGK
Ponceau
WB: αMYC
WT
nse3-1
150
37
KD
Smc5-MYC
αPGK
Ponceau

Supplemantry Figure 3, Related to Figure 3.
a., b. Yeast two-Hybrid analysis between Smc5/6 (Nse1, Mms21, Nse3 Nse4, Nse6 and Smc5) as prey vectors and Cohibin components (a) Csm1 or (b) Lrs4 as bait vectors.

c. Smc5/6 components as preys (Nse1, Mms21, Nse3 Nse4, Nse6 and Smc5) with empty vector (pGAL-LexA) control represent background β-galactosidase activity units.

d., e. Western blots with (d) α-HA and (d) α-LexA shows the expression levels of proteins with their corresponding epitope tags from Yeast-two hybrid vectors after induction in Galactose-containing media in comparison with Glucose-containing media as control. Glu: Glucose; Gal: Galactose. The expression vectors are listed in Table S3.

f. Western blots with α-FLAG for $Smc6^{FLAG}$ in WT (JC 1595) and *nse3*-1 (3078) used for ChIP in Fig. 3f, 3g and α-MYC for $Smc5^{MYC}$ in WT (JC 3467) and *nse3*-1 (3483) used for ChIP in Supplementary Fig. 1a,b.

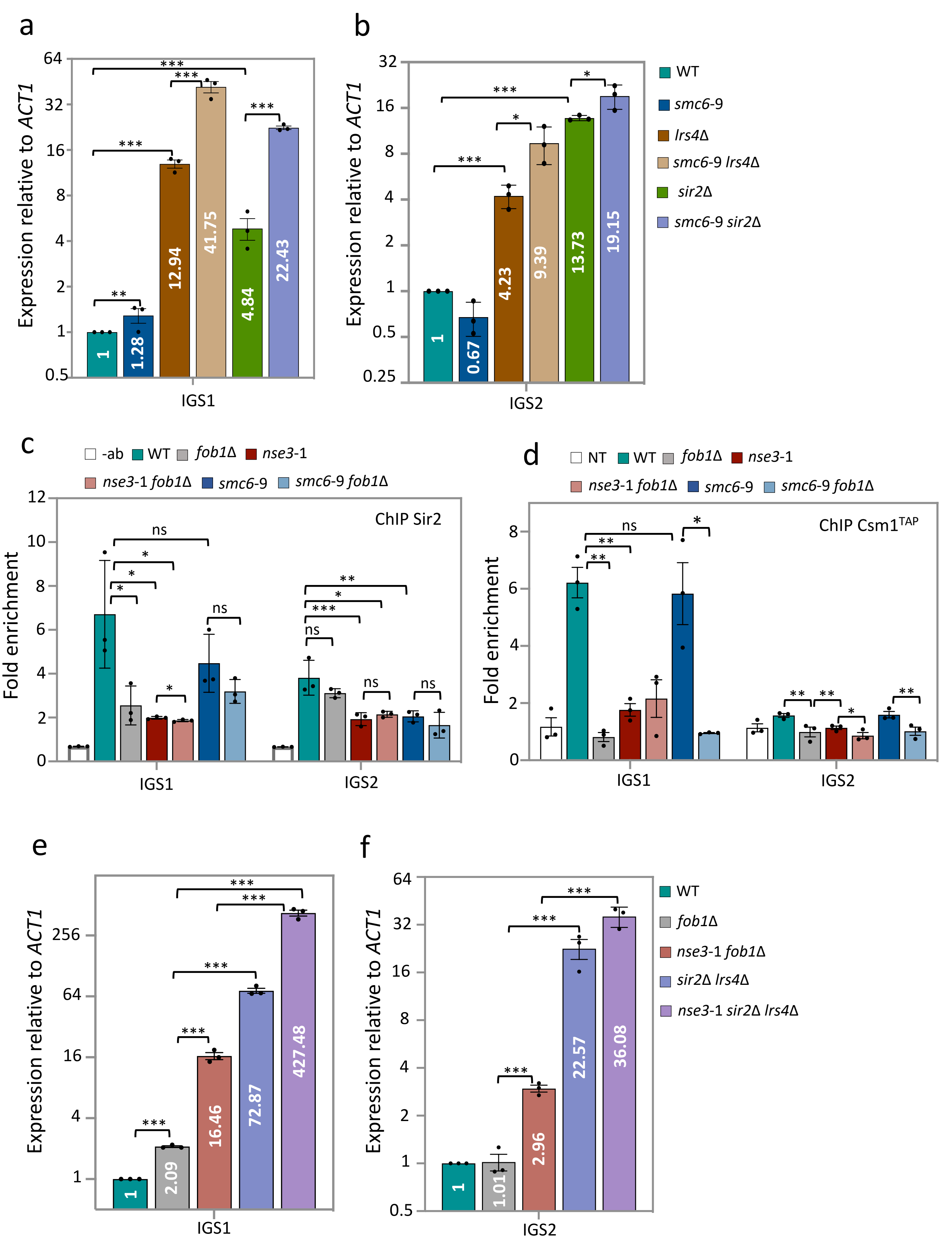
a
b
c
d
e
f
Expression relative to ACT1
IGS1
IGS2
1
1.28
12.94
41.75
4.84
22.43
0.67
4.23
9.39
13.73
19.15
WT
smc6-9
lrs4Δ
smc6-9 lrs4Δ
sir2Δ
smc6-9 sir2Δ
-ab
fob1Δ
nse3-1
nse3-1 fob1Δ
smc6-9 fob1Δ
NT
Fold enrichment
ChIP Sir2
ChIP Csm1TAP
ns
2.09
16.46
72.87
427.48
1.01
2.96
22.57
36.08
sir2Δ lrs4Δ
nse3-1 sir2Δ lrs4Δ

Supplemantry Figure 4, Related to Figures 3 and 4.
a., b. Transcription of (a) IGS1 and (b) IGS2 measured and represented as relative to WT cells after normalization to *ACT1* expression for WT (JC 471), *smc6*-9 (JC 1358), *lrs4*Δ (JC 3791), *smc6*-9 *lrs4*Δ (JC 4976), *sir2*Δ (JC 4648) and *smc6*-9 *sir2*Δ (JC 4978) strains**.**

c. Enrichment of Sir2 at IGS1 and IGS2 by ChIP with α-Sir2 in WT (JC 471), *fob1*Δ (4825), *nse3*-1(JC 3032), *nse3*-1 *fob1*Δ (JC 4595), *smc6*-9 (JC 1358) and *smc6*-9 *fob1*Δ (JC 4824). Fold enrichment is based on normalization to negative control region relative to no antibody control.

d. Enrichment of Csm1^{TAP} at IGS1 and IGS2 by ChIP with α-TAP in Non-tagged control (JC 470), WT (JC 4233), *fob1*Δ (JC 4937), *nse3*-1 (JC 4251), *nse3*-1 *fob1*Δ (JC 4973), *smc6*-9 (JC 4938) and *smc6*-9 *fob1*Δ (JC 4929) at IGS1 and IGS2. Fold enrichment is based on normalization to negative control region as described in Fig.1. Analysis was performed using at least three biological replicates. Statistical analysis is described in methods.

e., f. Transcription of (e) IGS1 and (f) IGS2 measured and represented as relative to WT cells after normalization to *ACT1* expression for WT (JC 471), *fob1*Δ (JC 4825), *nse3*-1 *fob1*Δ (JC 4595), *sir2*Δ *lrs4*Δ (JC 4979), *nse3*-1 *sir2*Δ *lrs4*Δ (JC 4980) strains**.**

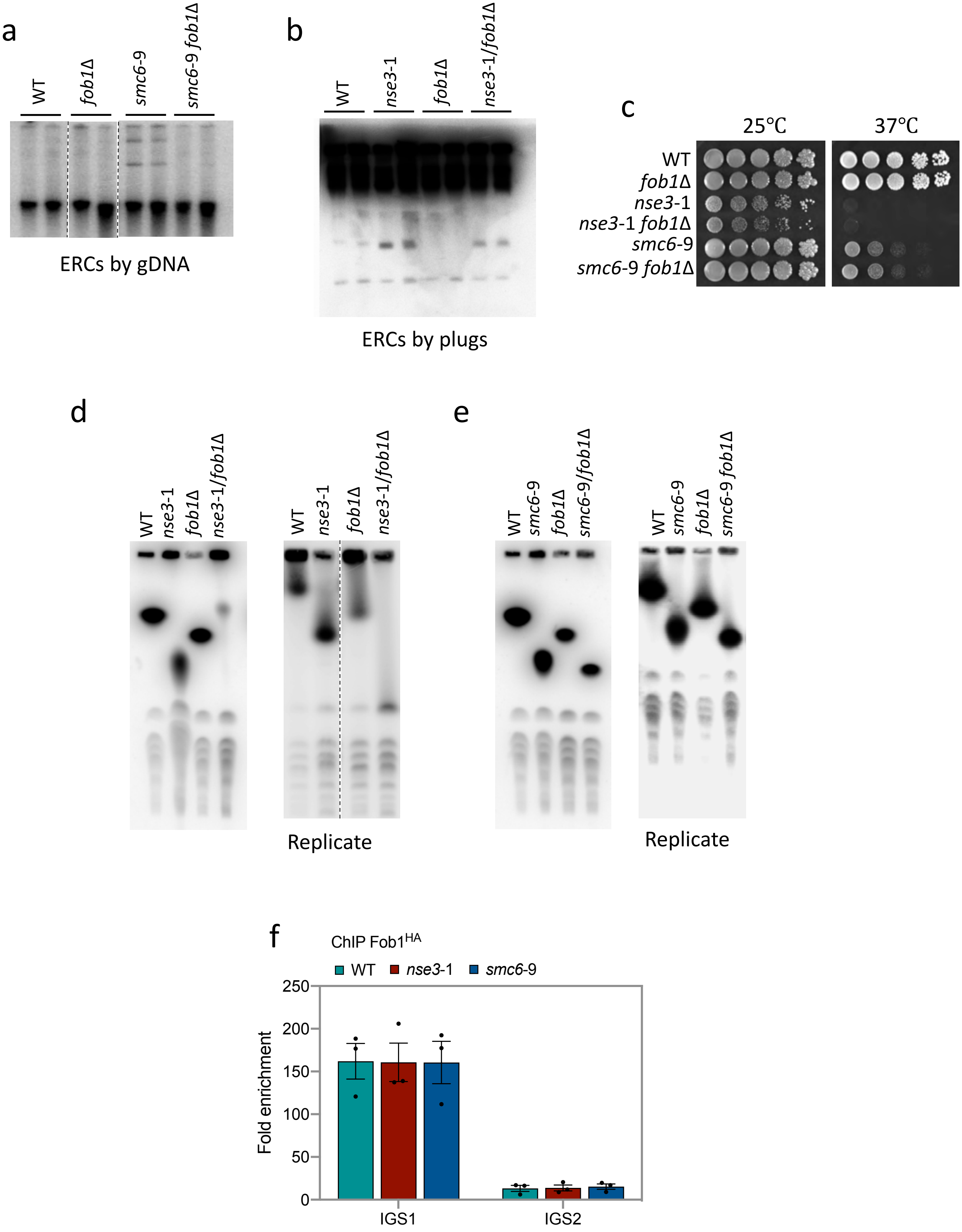
a
WT
fob1Δ
smc6-9
smc6-9 fob1Δ
ERCs by gDNA
b
WT
nse3-1
fob1Δ
nse3-1/fob1Δ
ERCs by plugs
c
25°C
37°C
WT
fob1Δ
nse3-1
nse3-1 fob1Δ
smc6-9
smc6-9 fob1Δ
d
WT
nse3-1
fob1Δ
nse3-1/fob1Δ
Replicate
e
WT
smc6-9
fob1Δ
smc6-9/fob1Δ
smc6-9 fob1Δ
Replicate
f
ChIP Fob1HA
WT
nse3-1
smc6-9
Fold enrichment
0
50
100
150
200
250
IGS1
IGS2

Supplementary Fig. 5, Related to Figure 4.

a. ERCs for WT (JC 470, JC 471), *fob1*Δ (JC 4825, JC 4825), *smc6*-9 (JC 1358, JC 3039) and *smc6*-9 *fob1*Δ (JC 4824, JC 4823) strains were visualized by running ≅ 10ug gDNA at low voltage for ≅ 24hours followed by southern blotting and probing for rDNA repeats.

b. ERCs for WT (JC 470, JC 471), *nse3*-1(JC 3032, JC 3084), *fob1*Δ (JC 4825, JC 4825) and *nse3*-1 *fob1*Δ (JC 5882, JC 5883) strains were visualized by running plugs containing 5x 10^7 cells/plug at low voltage for ≅ 24hours followed by southern blotting and probing for rDNA repeats.

c. Drop assay to check the temperature sensitivity of the strains - WT (JC 471), *fob1*Δ (JC 4825), *nse3*-1(JC 3032), *nse3*-1 *fob1*Δ (JC 4595), *smc6*-9 (JC 1358) and *smc6*-9 *fob1*Δ (JC 4824) on YPAD at 37°C.

d., e. rDNA repeats for WT (JC 471), *fob1*Δ (JC 4825), *nse3*-1(JC 3032), *nse3*-1 *fob1*Δ (JC 4595), *smc6*-9 (JC 1358) and *smc6*-9 *fob1*Δ (JC 4824) strains were visualized by PFGE, followed with southern blotting and probing for rDNA repeats. Independent replicate experiments were performed.

f. Enrichment of $Fob1^{HA}$ at IGS1 and IGS2 by ChIP with α-HA in WT (JC 5007), *nse3*-1 (JC 5008) and *smc6*-9 (JC 5010). Fold enrichment is based on normalization to negative control region (ZN). Analysis was performed using at least three biological replicates. Statistical analysis is described in methods.

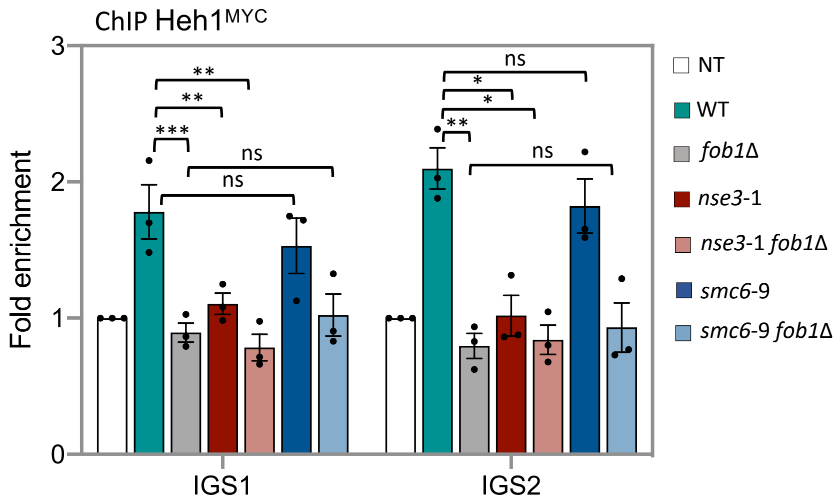

Supplementary Fig. 6.
Enrichment of Heh1^{MYC} at IGS1 and IGS2 by ChIP with α-MYC in non-tagged control (JC 470), WT (JC 4022), *fob1*Δ (JC 4940), *nse3*-1 (JC 4228), *nse3*-1 *fob1*Δ (JC 4975), *smc6*-9 (JC 4942) and *smc6*-9 *fob1*Δ (JC 4943) at IGS1 and IGS2. Fold enrichment is based on normalization to negative control region relative to non-tagged control. Analysis was performed using at least three biological replicates. Statistical analysis is described in methods.
